# *Opuntia ficus-indica* cladodes as a functional ingredient: bioactive compounds profile and their effect on antioxidant quality of bread

**DOI:** 10.1186/s12944-016-0397-y

**Published:** 2017-02-07

**Authors:** Lotfi Msaddak, Ola Abdelhedi, Amani Kridene, Mostafa Rateb, Lassaâd Belbahri, Emna Ammar, Moncef Nasri, Nacim Zouari

**Affiliations:** 1 Laboratory of Enzyme Engineering and Microbiology, Engineering National School of Sfax (ENIS), University of Sfax, Sfax, Tunisia; 2 Research Unit of Coastal and Urban Environments, ENIS, University of Sfax, Sfax, Tunisia; 3 School of Science and Sport, University of West of Scotland, Paisley, UK; 4 Laboratory of Soil Biology, University of Neuchatel, Neuchatel, Switzerland; 5 High Institute of Applied Biology of Medenine (ISBAM), University of Gabes, Medenine, Tunisia

**Keywords:** *Opuntia ficus*-*indica*, Cladodes, LC-HRESIMS, Flavonoids, Dough, Bread quality, Antioxidant

## Abstract

**Background:**

In the context of a balanced diet, the antioxidant-rich food consumption is a preventive way of many degenerative diseases. Consequently, improving the nutraceutical quality of traditional foods such as bakery products is an interesting approach. Considering the present consumer’s demand, cladodes from prickly pear that were traditionally used as a valuable food as well as in folk medicine for the treatment of several chronic diseases were investigated for their use in bread production to improve its functionality.

**Methods:**

Bioactive substances were determined by liquid chromatography-high resolution electrospray ionization mass spectrometry (LC-HRESIMS) analysis. Dough rheological properties were characterized by alveographic measurements. Bread antioxidant quality was evaluated by total phenolics content, DPPH• radical-scavenging, metal (Fe^2+^) chelating and Fe^3+^ reducing power determinations.

**Results:**

LC-HRESIMS analysis of the cladodes extract allowed the identification of 9 flavonoids, 2 phenolics, 1 alkaloid and 1 terpenoid compounds. Cladodes powder enrichment induced important modifications on the dough rheological parameters in terms of the extensibility (L) and deformation energy (W) decrease. Moreover, cladodes powder addition to bread resulted in a decrease in both crust and crumb colour parameters (L*, a* and b*). A 5% supplementation resulted in an increase of the bread yield and bread specific volume by 8.9 and 25%, respectively. Interestingly, Bread containing cladodes powder showed enhanced total phenolics content and antioxidant potential as compared to the control.

**Conclusions:**

Substitution of wheat flour by the cladodes powder at 5% level was optimal for improving the total phenolics content and the antioxidant potential of bread without having any negative effect on its sensory acceptability. Cladodes from *Opuntia ficus*-*indica* could be considered as a potential health-promoting functional ingredient in bakery products.

## Background

Functional food and nutraceuticals have the potential to become the future of primary prevention of many chronic diseases such as dyslipidaemia and cardiovascular diseases [1]. Besides, epidemiological studies have consistently shown that regular consumption of plant-based food is associated with reduced risk of chronic diseases related to oxidative stress. Therefore, incorporation of phytochemicals and particularly natural antioxidants into the conventional foods formulation may improve their nutraceutical potential. Bread made from refined wheat flour, a wide consumed product, is characterized by a low antioxidant potential and would be therefore an interesting support requiring the incorporation of functional supplements to improve its health benefits [2].

In the word, *Opuntia* plants are grown in several regions occupying around 100,000 ha, especially in South America, Mediterranean basin, Middle East and India. Annual production of cladodes (stems) from prickly pear (*Opuntia ficus*-*indica*) is varying from 30 to 80 t/ha, according to the region, meteorological conditions and plant variety [3]. Traditionally, the young cladodes have been consumed as vegetables in various forms, and they are claimed to be health-promoting food since they contain a great number of potentially active nutrients. Particularly, the high calcium, fibers and phenolics contents are worth to be mentioned in prickly pear cladodes [4, 5]. Dietary fibers and bioactive compounds are widely used as functional ingredients in processed foods. Therefore, cladodes powder was proposed as ingredient in milk-based drinks and breakfast cereals. Furthermore, it may be applied up to 20% as a thickening agent in vegetable soups and dessert gels [3]. Recently, cladodes powder was used as wheat flour substitute in the formulation of cookies containing high content of butter. Indeed, cladodes enrichment was found to be promising in terms of fat retention and oxidative damage reduction, resulting in more stable product [5]. Cactus cladodes are also widely used in folk medicine for the treatment of ulcers, wounds, diabetes and rheumatic pain; these uses are nowadays supported by scientific investigations [6]. The main health benefits of cladodes against chronic diseases are associated to their fibers and antioxidants. Particularly, cladodes were claimed to regulate both body mass and glycemia, to treat gastrointestinal disorders and to have an anti-hyperlipidemic effect [6]. It was suggested that soluble fibers increased stool bulking that accelerate peristaltic contractions and reduced the transit duration through the colon [3]. Recently, Uebelhack et al. [7] reported that the administration of tablets containing 500 mg of cactus fibers by healthy subjects increased fecal fat excretion without any adverse effect noticeable during the study period. Moreover, the cactus hypoglycemic effect was explained by its richness in viscous fibers that thicken the intestinal tract contents and may attenuate the sugar absorption. Although the *Opuntia* cladodes are traditionally used as a valuable healthy nutrient, they are scarcely used in modern nutrition. At the authors’ knowledge, few studies have focused on the bread enrichment with green parts of vegetables. Therefore, in the present work, *O. ficus-indica* cladodes were used as a new functional ingredient for enhancing nutraceutical properties of wheat bread. Cladodes were analyzed by Liquid Chromatography-High Resolution Electrospray Ionization Mass Spectrometry (LC-HRESIMS) technique in order to identify the bioactive compounds frequently associated with antioxidant activity. Then, the effects of cladodes powder substitution to wheat flour on dough alveographic properties as well as physical, antioxidant and sensory bread characteristics were assessed.

## Methods

### Preparation of cladodes powder

Cladodes from *Opuntia ficus*-*indica* f. *inermis* (spineless cladodes) were collected from the area of Gabes (Tunisia). Cladodes were washed, cut and dried in convection oven at 50 °C during 6 h (Polin A511088/AL/3125, Verona, Italy). The dried products were ground in a spice grinder (Black & Decker CBG100S Smartgrind, Maryland, USA), sieved through 250 μm sieve and then the obtained powder was stored at 25 °C before use.

### LC-HRESIMS analysis

The cladodes powder (25 g) was extracted by maceration using 250 ml of ethanol during 24 h. The solvent was then evaporated under vacuum and the residual solvent was removed by flushing with nitrogen. One hundred mg of cladodes extract was dissolved in 100 ml of 10% methanol, filtered through 0.45 μm filter, and then 1 ml was transferred into LC-MS vials. Reversed-phase column (Pursuit XRs ULTRA 2.8, C18, 100 mm × 2 mm i.d., Agilent Technologies, UK) and a diode array detector were used to carry out HPLC analyses. Twenty μl of the sample were injected into the column at a temperature set at 30 °C. The Mobile phases consisted of 0.1% formic acid in water (A) and 0.1% formic acid in methanol (B). A gradient program was used for separation at a flow rate of 1 ml/min. Mobile phases consisted of an initial composition of 100% solvent A, with a gradient of 100% solvent B over 20 min, hold at 100% solvent B for 5 min and 100% solvent A for 25 min. The drying gas flow rate was 1 ml/min at 320 °C. The mass spectrometer (MS) was operated in the positive ion mode in a mass range of 100–2000 m/z. High resolution mass spectral data were obtained on a Thermo Instruments ESI-MS system (LTQ XL/LTQ Orbitrap Discovery, UK) connected to a Thermo Instruments HPLC system (Accela PDA detector, Accela PDA autosampler and Accela Pump).

### Alveographic properties of dough

The dough alveographic properties were measured by an alveograph (Chopin alveograph MA 82, Tripette et Renaud, Villeneuve La Garenne, France) using the standard method [8]. The studied samples were wheat flour (control) and flour blends containing a mixture of wheat flour and cladodes powder in the ratios (m/m): 97.5/2.5 (formulation 1: F1), 95/5 (formulation 2: F2), 92.5/7.5 (formulation 3: F3), and 90/10 (formulation 4: F4). The following alveographic parameters (P, L and W) were automatically recorded by a computer software program. The maximum overpressure (P) needed to blow the dough bubble indicated the dough resistance to deformation or its tenacity. This parameter was related to the gluten quality and quantity as well as to its ability to absorb water. The average abscissa (L) at bubble rupture indicated the dough extensibility or the ability of the gluten to hold the gas. The configuration ratio (P/L) indicated the dough balance between the tenacity and the extensibility. The deformation energy (W) represented an index of dough strength.

### Bread elaboration

Flat bread was prepared in a local pastry industry (Société Pâtisserie-Masmoudi, Sfax, Tunisia). The standard bread formulation is consisted of: 1 kg wheat flour, 435 g water, 90 g olive oil, 33 g whole egg, 23 g NaCl, 8 g dried yeast (La Pâtissière, Tunisia) and 1.7 g Na_2_CO_3_. The wheat flour chemical composition expressed in g/100 g, was as follows: starch, 71.74; water, 13.40; proteins, 10.27; total fibers, 0.93 and fat, 0.77 as determined by a multipurpose analyzer (MPA) spectrometer (Bruker Optics, Wissembourg, France). Breads with variable rates of cladodes powder were made from wheat flour (control) and blends containing 25 g (F1), 50 g (F2), 75 g (F3) and 100 g (F4) of cladodes powder per 1 kg wheat flour substitution basis. The added water for F1, F2, F3 and F4 was 445, 455, 465 and 475 g, respectively. The yeast was dissolved in warm water (35 °C) and the resulting solution was added to the dry ingredients and finally the olive oil was added. The mixture was blended manually for 10 min and the resulting dough was fermented for 90 min at 30 °C. The dough circles of 50 mm diameter and 5 mm thin were shaped and placed on proofing trays for 2 h before baking, which was conducted at 180 °C for 10 min. Finally, the flat breads were cooled to room temperature and then stored at −18 °C in plastic bags.

### Physical properties of bread

Bread volume (cm^3^) was determined by the rapeseed displacement method and bread specific volume (cm^3^/g) was measured as bread volume divided by bread mass. Masse of dough or bread was measured using a precision balance. The bread yield and the mass loss representing the baking loss were calculated using Eqs. (1) and (2), respectively.

1 $$ \mathrm{Bread}\ \mathrm{yield}\ \left(\mathrm{g}/100\;\mathrm{g}\ \mathrm{flour}\right)=\frac{\mathrm{Bread}\ \mathrm{mass}\ }{\mathrm{Wheat}\ \mathrm{flour}\ \mathrm{mass}}\times 100 $$

2 $$ \mathrm{Mass}\ \mathrm{loss}\ \left(\%\right)=\frac{\left(\mathrm{Dough}\ \mathrm{mass}\hbox{--} \mathrm{Bread}\ \mathrm{mass}\right)\ }{\mathrm{Dough}\ \mathrm{mass}}\times 100 $$

Colour parameters (lightness L*, redness a* and yellowness b*) of crust and crumb were determined using a colour flex spectrocolorimeter (Hunter Associates Laboratory Inc., Reston, VA). L* value indicates the lightness, 0–100 representing dark to light, a* value gives the degree of the green–red colour, with a higher positive a* value indicating more red. The b* value indicates the degree of the blue–yellow colour, with a higher positive b* value indicating more yellow.

### Total phenolics content and antioxidant activity of bread

Total phenolics content, DPPH• radical-scavenging, metal (Fe^2+^) chelating and Fe^3+^ reducing power were measured in bread ethanolic extract as previously described [9, 10]. Ten g of bread sample were homogenized with 100 ml ethanol for 24 h at ambient temperature using an orbital shaker at stirring speed of 200 rpm. After filtration, the obtained extract was recovered and kept in the dark at 4 °C until further analyses. Total phenolics were expressed as mg gallic acid equivalents (GAE)/100 g of bread. Results of DPPH• radical-scavenging and metal (Fe^2+^) chelating assays are presented by IC_50_ values, defined as the extract concentration needed to scavenge 50% of DPPH• and to chelate 50% of Fe^2+^, respectively. In the reducing power assay, the presence of antioxidants in the sample would reduce the Fe^3+^ to Fe^2+^, which was monitored by measuring the Perl’s Prussian blue (Fe_4_[Fe(CN)_6_]_3_) formation at 700 nm. The reducing power (A_700_, absorbance at 700 nm) was determined at 2 mg/ml of bread extract.

### Sensory evaluation

The sensory properties (texture, colour, odour, taste and overall acceptability) of fresh prepared breads were evaluated according to the method of Murray et al. [11] by sixty panelists. A five-point hedonic scale was used for each attribute, where 5: like very much, 4: like moderately, 3: neither like nor dislike, 2: dislike moderately and 1: dislike very much.

### Statistical analysis

All analytical determinations were performed in triplicate. One-way analysis of variance was conducted using the SPSS software for Windows™ (version 17, SPSS Inc., Chicago, IL, USA). Duncan’s multiple range test (*p* < 0.05) was used to compare the averages responses between treatments.

## Results and discussion

### Phytochemical analysis of cladodes from *Opuntia ficus*-*indica*

The results for the phytochemical profile of the *O. ficus*-*indica* cladodes were presented in Table 1. In the present study, liquid chromatography-high resolution electrospray ionization mass spectrometry (LC-HRESIMS) analysis of the ethanolic extract of *O. ficus*-*indica* cladodes resulted in the identification of 13 compounds. These compounds are divided into 9 flavonoids, 2 phenolics, 1 alkaloid and 1 terpenoid compounds (Table 1). These results confirmed our previous findings, suggesting that *O. ficus*-*indica* cladodes were an important source of phenolics (2.48 g gallic acid equivalent/100 g DM) and flavonoids (1.06 g quercetin equivalent/100 g DM) [5]. Table 1 shows that some compounds such as quercetin, kaempferol, isorhamnetin 3-*O*-glucoside, isorhamnetin 3-*O*-neohesperidoside, coumaric acid and zataroside-A were previously reported in *O. ficus-indica* cladodes [12]. Whereas, in the present study further compounds (kaempferol and quercetin derivatives, isorhamnetin 3,3′,4′,5,7-pentahydroxy-flavanone, indicaxanthin and β-sitosterol) were also identified in *O. ficus-indica* cladodes (Table 1). A survey of the literature shows that some of these identified compounds had potent antioxidant potential. As a matter of fact, the IC_50_ values relative to the DPPH• radical-scavenging activities of kaempferol, quercetin, isorhamnetin and quercetin 3-*O*-glucoside were 1.7, 2.9, 5.0 and 10.5 μg/ml, respectively [13–16]. In Addition to its antioxidant potential, the flavonol quercetin is one of the most abundant dietary flavonoids that present diverse biological properties such as antiproliferative and anticarcinogenic activities [17]. Furthermore, Bouhlel et al. [18] reported that isorhamnetin 3-*O*-neohesperidoside was a potent inhibitor of xanthine oxidase and superoxide anion scavenger, which suggest that this flavonoid is able to protect cells against the consequences of oxidative stress. Indicaxanthin, a main betalain pigment in prickly pear fruit, is also a powerful antioxidant since it is more effective than 6-hydroxy-2,5,7,8-tetramethylchroman-2-carboxylic acid (Trolox) in scavenging the 2,2′-azinobis(3-ethylbenzothiazoline-6-sulfonic acid) radical cation (ABTS•+) [19]. Betalains from plants are presently gaining popularity for use as natural colorants in the food industry [20]. Table 1 shows that terpenoids were represented by β-sitosterol. Interestingly, several works suggest a protective role of this phytosterol against colon, prostate and breast cancers [21, 22]. Moreover, Baskar et al. [22] showed that β-sitosterol has chemopreventive potential through its potent radical scavenging capacity, with relatively low toxicity to normal cells.

**Table 1:** Liquid Chromatography-High Resolution Electrospray Ionization Mass Spectrometry (LC-HRESIMS) analysis of the cladodes ethanolic extract

Suggested compounds^a^	Accurate mass	Molecular formula^b^
Flavonoids		
Quercetin	303.04953	C_15_H_10_O_7_
Quercetin 3-*O*-glucoside	465.10225	C_21_H_20_O_12_
Kaempferol	287.05591	C_15_H_10_O_6_
Kaempferol 3-*O*-glucoside	449.10744	C_21_H_20_O_11_
Kaempferol 3-*O*-rutinoside	595.16535	C_27_H_30_O_15_
Isorhamnetin	317.06581	C_16_H_12_O_7_
Isorhamnetin 3-*O*-glucoside	479.11880	C_22_H_22_O_12_
Isorhamnetin 3-*O*-neohesperidoside	625.17671	C_28_H_32_O_16_
3,3′,4′,5,7-Pentahydroxy-flavanone	305.06588	C_15_H_12_O_7_
Phenolics		
p-Coumaric acid	165.05427	C_9_H_8_O_3_
Zataroside-A	329.15954	C_16_H_24_O_7_
Alkaloid		
Indicaxanthin	309.10851	C_14_H_16_N_2_O_6_
Terpenoid		
β-Sitosterol	415.39396	C_29_H_50_O

^a^ The compounds are suggested according to the Dictionary of Natural Products (DNP 23.1, 2015 on DVD) and the characteristic fragmentation pattern; ^b^ The formulas were deduced from the quasi molecular ion peak [M + H]^+^

Many studies have revealed a positive correlation between nutraceuticals and functional food, and reduced risk of diseases associated with oxidative stress such as cardiovascular diseases, cancers, diabetes as well as neurodegenerative diseases [1, 6]. Thus, the consumption of food products enriched with prickly pear cladodes would potentially provide antioxidant properties and consequently health benefits.

### Alveographic characteristics and yield of the dough

Table 2 shows that a partial substitution (from 2.5 to 10%) of wheat flour by the cladodes powder induced significant (*p* < 0.05) modifications on the dough rheological parameters (P, L and W). Indeed, when the substitution level increased, a significant (*p* < 0.05) reduction in the extensibility (L) and in the deformation energy (W) was observed, indicating that high substitution level opposed to the dough development. By comparing the control sample and the F4 formulation containing 10% of cladodes powder, (L) and (W) values decreased by 65.1 and 23.1%, respectively. Nevertheless, the tenacity of the dough (P) significantly (*p* < 0.05) increased by 1.7 folds at 10% substitution level, which might be a probable consequence of poor gluten hydration. Table 2 also shows that the noticed effect on (P) and (L) parameters became evident in the (P/L) ratio increase, indicating inextensible dough. Similar results were found by Borchani et al. [23], who reported an important increase in the tenacity (P), and a reduction in the deformation energy (W) and extensibility (L) of the dough containing different levels of date flesh fibers concentrate (from 0.5 to 3%). The present results could be mainly explained by the high water holding capacity of cladodes powder. In fact, in our previous work, cladodes powder was found to contain a high content of dietary fibers (28.84 g/100 g DM) and presented important hydration properties described by swelling index (6.31 cm^3^/g) and water holding capacity (795 g/100 g DM) [5]. Furthermore, interactions between fibers and gluten could affect the gluten network structure. Therefore, gluten proteins were not well hydrated and developed in order to form a viscoelastic network able to retain the fermentation gas.

**Table 2:** Effect of cladodes powder on dough alveographic properties and bread characteristics

Parameters	Level of cladodes powder substitution				
	Control (0%)	F1 (2.5%)	F2 (5%)	F3 (7.5%)	F4 (10%)
Dough					
P (mm H_2_O)	82.3 ± 0.6^d^	117.6 ± 1.3^c^	124.5 ± 0.7^b^	132.3 ± 5.6^b^	142.0 ± 1.7^a^
L (mm)	68.0 ± 1.6^a^	36.0 ± 1.4^b^	36.5 ± 2.1^b^	28.3 ± 2.1^bc^	23.7 ± 0.6^c^
P/L	1.20 ± 0.03^d^	3.20 ± 0.20^c^	3.30 ± 0.10^c^	4.80 ± 0.20^b^	5.90 ± 0.05^a^
W (10^−4^ J)	193.7 ± 1.5^a^	198.0 ± 2.8^a^	176.8 ± 3.8^b^	180.0 ± 4.2^b^	152.0 ± 5.2^c^
Bread					
Mass loss (%)	24.2 ± 0.3^a^	23.4 ± 0.2^a^	22.5 ± 0.3^b^	21.7 ± 0.1^c^	21.0 ± 0.1^d^
Yield (g/100 g flour)	120.0 ± 0.2^e^	125.1 ± 0.5^d^	130.7 ± 0.6^c^	136.6 ± 0.1^b^	142.6 ± 0.4^a^
Volume (cm^3^)	9.9 ± 0.1^d^	11.8 ± 0.4^c^	14.2 ± 0.3^a^	13.3 ± 0.4^b^	10.6 ± 0.9^cd^
Specific volume (cm^3^/g)	1.20 ± 0.03^d^	1.30 ± 0.10^c^	1.50 ± 0.03^b^	1.60 ± 0.04^a^	1.30 ± 0.10^c^

^a, b, c, d, e^ Values with the same superscript letters in the same line are non-significant at *p* < 0.05

Table 2 shows that the bread mass loss, related to the water loss after baking, was significantly (*p* < 0.05) affected by cladodes level incorporation. In fact, it was reduced by 13.2% at 10% substitution level (F4). Besides, cladodes powder addition significantly (*p* < 0.05) increased the bread yield that rose by 18.8% at 10% substitution level (F4). Bread yield increase could be attributed to the wheat flour substitution by cladodes powder, as it was expressed on wheat flour basis. Moreover, obtained results could be also explained by the high water retention of the fortified dough, since addition of cladodes powder required more water during dough elaboration. Similar results were also found by Borchani et al. [23], who evidenced a dough yield increase of 4.3% at 3% flour substitution level with date flesh fibers. However, these authors showed that the bread mass loss was not significantly (*p* > 0.05) affected by date flesh fibers enrichment (from 0.5 to 3%). Consequently, the wheat flour substitution by cladodes powder and the increase of the dough absorption were effective in increasing bread yield, which was important considering the economical aspect.

### Effects of cladodes powder integration on bread quality

#### Physical properties of bread

**Fig. 1:**
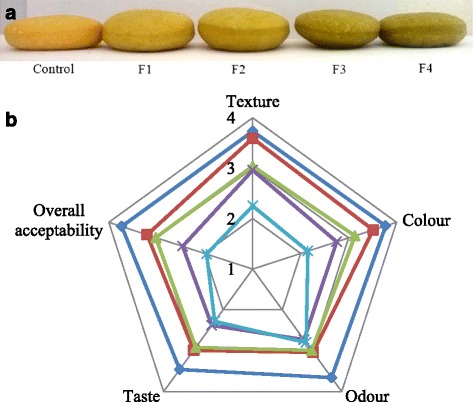
**a** Bread prepared with 2.5% (F1), 5% (F2), 7.5% (F3) and 10% (F4) of cladodes powder from prickly pear. The control represented the product without enrichment. **b** Sensory evaluation of the formulated breads. The attributes were evaluated using a five-point hedonic scale, where 5: like very much, 4: like moderately, 3: neither like nor dislike, 2: dislike moderately and 1: dislike very much

Table 2 shows a significant (*p* < 0.05) increase in the bread volume as well as in the specific volume that reached maximum values in F2 and F3 formulations (Fig. 1a). In fact, the average specific volume increased from 1.2 cm^3^/g (control bread) to 1.6 cm^3^/g at 7.5% substitution level (F3) and then it decreased to 1.3 cm^3^/g at 10% substitution level (F4). Similarly, Ayadi et al. [4] demonstrated that the cake specific volume increased by 20% at 10% substitution level of wheat flour by spineless cladodes powder, while it decreased by 15% at 20% substitution level. Nevertheless, further studies conducted on breads fortified by other agro-resources rich in fibers did not report any increase in specific volume as it was found in the present study. In fact, some studies reported a significant (*p* < 0.05) decline in the loaf specific volume enriched by 3–9% lemon fibers or by 5% *Malva aegyptiaca* leaf powder [24, 25]. These authors suggest that insoluble fibrous material interfere with the gluten network formation and exhibit a destabilizing effect at the interfaces of the dough gas cells, which led to a decrease in the gas retention capacity [24, 25]. The different effects of plant materials enrichment on bread specific volume could be explained by the different techno-functional properties of their fibers and subsequently their interactions with the other dough components.

Table 3 shows the effects of cladodes powder substitution on the bread crust and crumb colours. A significant (*p* < 0.05) difference between control bread and samples enriched with cladodes powder was observed. Breads fortified by cladodes powder was found to be darker than the control product, as shown by the lower L* values for the crust and the crumb of the formulated breads. In addition, a* and b* values for both crust and crumb of enriched breads decreased with increasing the substitution level, this indicated that the colour initially yellowish declined to shift to a greenish one. This could be mainly explained by the richness of cladodes powder in chlorophyll and to other pigments. Although the cladodes powder addition resulted in a certain variation in the bread physical properties, such substitution could improve its nutritional value, due to the richness of prickly pear cladodes in beneficial phytochemicals.

**Table 3:** Colour characteristics of bread supplemented with cladodes powder

		L*	a*	b*
Crust colour	Control (0%)	62.91 ± 0.10^a^	3.53 ± 0.27^a^	20.24 ± 0.32^a^
	F1 (2.5%)	60.25 ± 0.55^b^	0.95 ± 0.06^b^	18.53 ± 0.62^b^
	F2 (5%)	58.58 ± 0.36^c^	0.30 ± 0.02^c^	16.44 ± 0.42^c^
	F3 (7.5%)	54.58 ± 0.19^d^	0.11 ± 0.01^d^	13.84 ± 0.47^d^
	F4 (10%)	53.55 ± 0.36^e^	0.10 ± 0.01^d^	13.45 ± 0.27^d^
Crumb colour	Control (0%)	66.31 ± 0.25^a^	0.94 ± 0.13^a^	17.06 ± 0.33^a^
	F1 (2.5%)	62.66 ± 0.40^b^	−0.67 ± 0.08^b^	16.98 ± 0.39^a^
	F2 (5%)	60.08 ± 0.40^c^	−0.73 ± 0.05^b^	15.87 ± 0.36^b^
	F3 (7.5%)	57.13 ± 0.30^d^	−0.87 ± 0.05^c^	14.47 ± 0.18^c^
	F4 (10%)	55.78 ± 0.10^e^	−0.95 ± 0.06^c^	13.51 ± 0.10^d^

^a, b, c, d^ Values with same superscript letters in the same column are non-significant at *p* < 0.05

#### Total phenolics content and antioxidant activity

Table 4 shows the total phenolics content, DPPH• radical-scavenging, metal (Fe^2+^) chelating activity and Fe^3+^ reducing activities of fortified breads. Bread prepared with 100% wheat flour was attributed to the lowest total phenolics content (0.9 mg GAE/100 g of bread), DPPH• radical-scavenging activity (IC_50_ = 48.30 mg/ml), Fe^2+^ chelating activity (IC_50_ = 102.36 mg/ml) and Fe^3+^ reducing power (A_700_ = 0.02). Interestingly, wheat flour substitution by cladodes powder significantly (*p* < 0.05) enhanced the total phenolics content and consequently the antioxidant activity of the fortified breads. Indeed, Table 4 shows that at 10% substitution level, bread exhibited the highest total phenolics content (9.36 mg GAE/100 g of bread), DPPH• radical-scavenging activity (IC_50_ = 1.25 mg/ml), Fe^2+^ chelating activity (IC_50_ = 5.03 mg/ml) and Fe^3+^ reducing power (A_700_ = 2.79). A comparable trend was also reported for wheat bread enriched with plant materials such as pomegranate peels, onion skin, grape seed or mallow powder, which improved its antioxidant potential [2, 25]. Similarly, Msaddak et al. [5] reported that cladodes powder supplementation in cookies enhanced their antioxidant potential as well as their stabilization against oxidative damage. The highly potent antioxidants compounds such as flavonoids, phenolics and alkaloid identified in cladodes (Table 1) may contribute individually or synergistically to the antioxidant activity of the fortified bread and thereby reinforced its nutraceutical effect. Thus, bread enrichment with cladodes from prickly pear seems to be a very easy and a cheap way for improving food quality. However, bread enrichment by plant raw materials could result to undesirable sensory attributes. Therefore, a compromise between nutritional value and sensory quality was investigated.

**Table 4:** Total phenolics and antioxidant activity of bread enriched with cladodes powder

	Level of cladodes powder substitution				
	Control (0%)	F1 (2.5%)	F2 (5%)	F3 (7.5%)	F4 (10%)
Total phenolics	0.90 ± 0.01^a^	2.36 ± 0.01^b^	5.22 ± 0.02^c^	7.83 ± 0.04^d^	9.36 ± 0.06^e^
Scavenging activity	48.30 ± 0.02^a^	34.80 ± 0.01^b^	14.68 ± 0.04^c^	7.25 ± 0.10^d^	1.25 ± 0.01^e^
Fe^2+^ chelating activity	102.36 ± 2.39^a^	47.15 ± 4.00^b^	33.81 ± 2.10^c^	12.86 ± 1.73^d^	5.03 ± 0.11^e^
Fe^3+^ reducing power	0.02 ± 0.00^a^	0.36 ± 0.01^b^	0.76 ± 0.01^c^	1.23 ± 0.10^d^	2.79 ± 0.40^e^

^a, b, c, d, e^ Values with the same superscript letters in the same line are non-significant at *p* < 0.05; Total phenolics are expressed as mg gallic acid equivalents (GAE)/100 g of bread; DPPH• radical-scavenging and metal (Fe^2+^) chelating activities are presented as IC_50_ values (mg/ml); Fe^3+^ reducing power (A_700_, absorbance at 700 nm) is determined at 2 mg/ml of bread extract

#### Sensory properties

Sensory analysis was carried out by assessing texture, colour, odour, taste and overall acceptability of fresh prepared breads (Fig. 1b). Obtained results showed that the increase of the incorporated cladodes powder decreased the trend in average scores for all analyzed attributes. Indeed, textural hardness, an important sensory characteristic of the bread, remained acceptable (score ≥ 3.0) up to 7.5% substitution level (Fig. 1b). However, at 10% substitution level (F4) the bread texture was inacceptable (2.25 in F4 *vs* 3.73 in control) as was evidenced by the alveographic assay. The bread colour is also important parameter acting on its acceptability. The cladodes powder, rich in chlorophyll, gave greenish colour characteristic to the formulated breads especially for F3 and F4 (Fig. 1a). At 5% substitution level (F2) the colour score was acceptable (3.13 in F2 *vs* 3.77 in control), while it decreased at the highest substitution levels. Likewise, at the highest substitution levels (F3 and F4) the odour and the taste scores were not acceptable. In terms of overall acceptability, the bread containing 5% of cladodes powder (F2) remained acceptable since the obtained mean score for the overall acceptability was 3.02 (Fig. 1b). Similarly to cladodes incorporation, Dziki et al. [2] reported that wheat flour substitution up to 3–5% by herbal ingredients such as the onion skin, turmeric powder or green tea exhibited consumer satisfaction for the enriched bread.

## Conclusions

Cladodes from prickly pear were traditionally used as a valuable food as well as in folk medicine. However, the vegetative parts of *Opuntia* cactus were scarcely used in the modern nutrition. In the present study, cladodes powder was analyzed for its bioactive compounds profile and then valorized by its integration in bread production to improve its functionality. High resolution mass spectrometric analysis of the cladodes allowed the identification of 13 compounds, among them many compounds had potent antioxidant potential. Cladodes powder incorporation into wheat flour modified the dough rheological parameters and thereby the bread physical properties. The obtained results showed that up to 5% of substitution level, cladodes powder could be included in a wheat bread formulation without altering its physical and sensory properties. Furthermore, such incorporation significantly (*p* < 0.05) enhanced the bread yield as well as its total phenolics content and antioxidant potential. Therefore, cladodes from prickly pear could be regarded as a potential health-promoting functional ingredient in bakery products.

## References

[1] Scicchitano P, Cameli M, Maiello M, Modesti PA, Muiesan ML, Novo S, Palmiero P, Saba PS, Pedrinelli R, Ciccone MM (2014). Nutraceuticals and dyslipidaemia: Beyond the common therapeutics. J Funct Foods.

[2] Dziki D, Rózyło R, Gawlik-dziki U, Świeca M (2014). *Current* trends in the enhancement of antioxidant activity of wheat bread by the addition of plant materials rich in phenolic compounds. Trend Food Sci Technol.

[3] Stintzing FC, Carle R (2005). Cactus stems (*Opuntia* spp.): A review on their chemistry, technology, and uses. Mol Nutr Food Res.

[4] Ayadi MA, Abdelmaksoud W, Ennouri M, Attia H (2009). Cladodes from *Opuntia ficus indica* as a source of dietary fiber: effect on dough characteristics and cake making. Ind Crop Prod.

[5] Msaddak L, Siala R, Fakhfakh N, Ayadi MA, Nasri M, Zouari N (2015). Cladodes from prickly pear as a functional ingredient: Effect on fat retention, oxidative stability, nutritional and sensory properties of cookies. Int J Food Sci Nutr.

[6] Osuna-Martínez U, Reyes-Esparza J, Rodríguez-Fragoso L (2014). Cactus (*Opuntia ficus-indica*): a review on its antioxidants properties and potential pharmacological use in chronic diseases. Nat Prod Chem Res.

[7] Uebelhack R, Busch R, Alt F, Beah ZM, Chong PW (2014). Effects of cactus fiber on the excretion of dietary fat in healthy subjects: a double blind, randomized, placebo-controlled, crossover clinical investigation. Curr Ther Res Clin Exp.

[8] AACC (2000). Approved methods of the American Association of Cereal Chemists, method 54-30 (alveogrpahic analysis).

[9] Yildirim A, Mavi A, Kara AA (2001). Determination of antioxidant and antimicrobial activities of *Rumex crispus* L. extracts. J Agric Food Chem.

[10] Zouari N, Ayadi I, Fakhfakh N, Rebai A, Zouari S (2012). Variation of chemical composition of essential oils in wild populations of *Thymus algeriensis* Boiss. et Reut., a North African endemic species. Lipids Health Dis.

[11] Murray JM, Delahunty CM, Baxter IA (2001). Descriptive sensory analysis: past, present and future. Food Res Int.

[12] Saleem M, Kim HJ, Han CK, Jin C, Lee YS (2006). Secondary metabolites from *Opuntia ficus-indica* var. Saboten. Phytochemistry.

[13] Hirano R, Sasamoto W, Matsumoto A, Itakura H, Igarashi O, Kondo K (2001). Antioxidant ability of various flavonoids against DPPH radicals and LDL oxidation. J Nutr Sci Vitaminol.

[14] Cos P, Rajan P, Vedernikova I, Calomme M, Pieters L, Vlietinck AJ, Augustyns K, Haemers A, Berghe DV (2002). In vitro antioxidant profile of phenolic acid derivatives. Free Radic Res.

[15] Leu CH, Li CY, Yao X, Wu TS (2006). Constituents from the leaves of *Phellodendron amurense* and their antioxidant activity. Chem Pharm Bull.

[16] Pengfei L, Tiansheng D, Xianglin H, Jianguo W (2009). Antioxidant properties of isolated isorhamnetin from the sea buckthorn marc. Plant Food Hum Nutr.

[17] Valerio LG, Kepa JK, Pickwell GV, Quattrochi LC (2001). Induction of human NAD(P)H:quinone oxidoreductase (NQO1) gene expression by the flavonol quercetin. Toxicol Lett.

[18] Bouhlel I, Limem I, Skandrani I, Nefatti A, Ghedira K, Dijoux-Franca MG, Leila CG (2010). Assessment of isorhamnetin 3-*O*-neohesperidoside from *Acacia salicina*: Protective effects toward oxidation damage and genotoxicity induced by aflatoxin B1 and nifuroxazide. J Appl Toxicol.

[19] Gengatharan A, Dykes GA, Choo WS (2015). Betalains: natural plant pigments with potential application in functional foods. LWT-Food Sci Technol.

[20] Butera D, Tesoriere L, Di Gaudio F, Bongiorno A, Allegra M, Pintaudi AM, Kohen R, Livrea MA (2002). Antioxidant activities of Sicilian prickly pear (*Opuntia ficus-indica*) fruit extracts and reducing properties of its betalains: betanin and indicaxanthin. J Agric Food Chem.

[21] Awad AB, Chan KC, Downie AC, Fink CS (2000). Peanuts as a source of beta-sitosterol, a sterol with anticancer properties. Nutr Cancer.

[22] Baskar AA, Ignacimuthu S, Paulraj GM, Al Numair KS (2010). Chemopreventive potential of beta-Sitosterol in experimental colon cancer model-an In vitro and In vivo study. BMC Complement Altern Med.

[23] Borchani C, Masmoudi M, Besbes S, Attia H, Deroanne C, Blecker C (2011). Effect of date flesh fiber concentrate addition on dough performance and bread quality. J Texture Stud.

[24] Fu JT, Chang YH, Shiau SY (2015). Rheological, antioxidative and sensory properties of dough and Mantou (steamed bread) enriched with lemon fiber. LWT-Food Sci Technol.

[25] Fakhfakh N, Jdir H, Jridi M, Rateb M, Belbahri L, Ayadi MA, Nasri M, Zouari N (2017). The mallow, *Malva aegyptiaca* L. (*Malvaceae*): phytochemistry analysis and effects on wheat dough performance and bread quality. LWT-Food Sci Technol.

